# Blue light at night produces stress-evoked heightened aggression by enhancing brain-derived neurotrophic factor in the basolateral amygdala

**DOI:** 10.1016/j.ynstr.2023.100600

**Published:** 2023-12-15

**Authors:** Zhenlong Li, Chau-Shoun Lee, Si Chen, Benyu He, Xinya Chen, Hsien-Yu Peng, Tzer-Bin Lin, Ming-Chun Hsieh, Cheng-Yuan Lai, Dylan Chou

**Affiliations:** a School of Basic Medical Sciences, Zhuhai Campus of Zunyi Medical University, Zhuhai, Guangdong, China; b Department of Medicine, MacKay Medical College, New Taipei, Taiwan; c Department of Psychiatry, MacKay Memorial Hospital, Taipei, Taiwan; d School of Bioengineering, Zhuhai Campus of Zunyi Medical University, Zhuhai, Guangdong, China; e Institute of Biomedical Sciences, MacKay Medical College, New Taipei, Taiwan; f Institute of New Drug Development, College of Medicine, China Medical University, Taichung, Taiwan; g Cell Physiology and Molecular Image Research Center, Wan Fang Hospital, Taipei Medical University, Taipei, Taiwan; h Department of Physiology, School of Medicine, College of Medicine, Taipei Medical University, Taipei, Taiwan

**Keywords:** Blue light at night, Aggressive behavior, Basolateral amygdala, Brain-derived neurotrophic factor, Long-term potentiation, Long-term depression

## Abstract

Light is an underappreciated mood manipulator. People are often exposed to electronic equipment, which results in nocturnal blue light exposure in modern society. Light pollution drastically shortens the night phase of the circadian rhythm. Preclinical and clinical studies have reported that nocturnal light exposure can influence mood, such as depressive-like phenotypes. However, the effects of blue light at night (BLAN) on other moods and how it alters mood remain unclear. Here, we explored the impact of BLAN on stress-provoked aggression in male Sprague‒Dawley rats, focusing on its influence on basolateral amygdala (BLA) activity. Resident-intruder tests, extracellular electrophysiological recordings, and enzyme-linked immunosorbent assays were performed. The results indicated that BLAN produces stress-induced heightened aggressive and anxiety-like phenotypes. Moreover, BLAN not only potentiates long-term potentiation and long-term depression in the BLA but also results in stress-induced elevation of brain-derived neurotrophic factor (BDNF), mature BDNF, and phosphorylation of tyrosine receptor kinase B expression in the BLA. Intra-BLA microinfusion of BDNF RNAi, BDNF neutralizing antibody, K252a, and rapamycin blocked stress-induced heightened aggressive behavior in BLAN rats. In addition, intra-BLA application of BDNF and 7,8-DHF caused stress-induced heightened aggressive behavior in naïve rats. Collectively, these results suggest that BLAN results in stress-evoked heightened aggressive phenotypes, which may work by enhancing BLA BDNF signaling and synaptic plasticity. This study reveals that nocturnal blue light exposure may have an impact on stress-provoked aggression. Moreover, this study provides novel insights into the BLA BDNF-dependent mechanism underlying the impact of the BLAN on mood.

## Highlights

• BLAN results in stress-induced heightened aggression.
• BLAN potentiates LTP and LTD in the BLA.
• BLAN produces stress-induced elevation of BLA BDNF signaling.
• Inhibiting BLA BDNF signaling blocks BLAN-dependent heightened aggression.
• Activating BLA BDNF signaling produces stress-induced heightened aggression.

## 1 Introduction

Day and night cycles on earth synchronize biological circadian rhythms that influence the physiology and health of living organisms, including body temperature, endocrine functions, metabolism, sleep and arousal, cognition, and mood (Vandewalle et al., 2009; LeGates et al., 2014; Walker et al., 2020; Tam et al., 2021). Additional illumination exposure to different periods of light and darkness may have distinct effects. Diurnal light therapy in humans is widely presumed to exert anti-depressive effects, whereas exposure to nocturnal light can produce opposite effects (Golden et al., 2005; Bais et al., 2016; Lam et al., 2016; Heo et al., 2017; An et al., 2020). Excess exposure to light at night (LAN), such as skylights or electronic devices, including smartphones, computers, tablets, and televisions before sleep, has been associated with a greater risk of depressive symptoms (Bedrosian and Nelson, 2013; Obayashi et al., 2013; Min and Min, 2018; Zielinska-Dabkowska, 2018; An et al., 2020; Walker et al., 2020). These contradictory findings suggest that the timing of light exposure is crucial. In addition to timing, the intensity of light seems to be another issue that needs to be studied (An et al., 2020; Walker et al., 2020). A full moon at night provides <1 lux (Gaston et al., 2013). Modern streets of cities in industrialized countries are lit by approximately 5–15 lux at night; moreover, the night-time light intensities in modern living rooms are approximately 100–300 lux (Gaston et al., 2013; Walker et al., 2020). The effects of light exposure timing and intensity remain largely unclear. The New World Atlas of Artificial Sky Brightness reported that >80% of the population worldwide and approximately 99% of individuals in America and Europe live under light-polluted skies (Falchi et al., 2016; Walker et al., 2020). Nocturnal light pollution has been recognized a major risk factor that influences human health, including obesity, breast cancer, and most importantly, mental health (Muscogiuri et al., 2022; Tancredi et al., 2022). Hence, an over-illuminated night-life raises concerns regarding its potential harmful impacts, particularly on mood (Bedrosian and Nelson, 2013).

In human society, aggression and violence represent both mental health and social problems, which are usually comorbidities of various neuropsychiatric diseases, including epilepsy, schizophrenia, depression, antisocial behavior, and autism (Nelson and Trainor, 2007; Lane et al., 2009; Comai et al., 2012). In rodent studies, aggressive behavior is thought to be an individual's intent to defeat someone, and has evolved as an adaptation to deal with competition (Nelson and Trainor, 2007). Pathologically escalated aggression includes a dramatic increase in biting events, shortened bite latencies, disregard for submissive signals by an opponent, and indiscriminate attack terrain (de Boer, 2018; Newman et al., 2018). Stressful events and social isolation are involved in the development of escalated aggression (Tsuda et al., 1988; Zelikowsky et al., 2018). Impulsively aggressive individuals may attack other individuals under stressful conditions (Chang and Gean, 2019). The animals suddenly experienced foot shock stress and exhibited aggressive phenotypes (Chang and Gean, 2019; Chang et al., 2021). Several brain regions, such as the amygdala (Marquez et al., 2013), medial prefrontal cortex (Newman et al., 2018), ventral hippocampus (Chang et al., 2015), hypothalamus (Lin et al., 2011), ventrolateral periaqueductal gray (Ye et al., 2019; Chou, 2020b, 2020a), dorsal raphe nucleus, and ventral tegmental area, have been suggested to contribute to animal aggression (Nelson and Trainor, 2007). Nevertheless, the influence of LAN on aggression and associated brain regional activity remains largely uninvestigated.

Most recently, a study reported that short- (such as blue light), but not long-wavelength LAN, increased the number of c-fos-positive cells in the basolateral nucleus of the amygdala (BLA) (Wang et al., 2023), revealing the potential impact of LAN on the BLA and perhaps on BLA-associated behavioral phenotypes, such as aggression. In the present study, we investigated the effects of blue light at night (BLAN) on BLA synaptic activity and plasticity-related brain-derived neurotrophic factor (BDNF) signaling, and explored its potential action on basal and stress-evoked aggression. The present study reported a novel finding on the potential impacts of BLAN on stress-provoked aggression and provided new insight into the fundamental understanding of the BLA BDNF-dependent mechanism behind nocturnal light-manipulated moods.

## 2 Materials and methods

### 2.1 Animals

Adult male Sprague‒Dawley (8 weeks of age) rats (BioLASCO Taiwan Co. Ltd., Taiwan) were used in the study. Four rats were group-housed in each cage in a temperature- (25 ± 1 °C) and humidity-controlled room on a 12 h light/dark cycle (lights on 06:00–18:00 h), with access to food and water provided *ad libitum*. Rats were acclimated in the animal research facility for at least one week before the experiments began. All behavioral procedures were performed during the light cycle between 10:00 and 15:00 h. All experimental procedures were conducted in accordance with the Animal Research: Reporting of In Vivo Experiments (ARRIVE) guidelines and approved by the Institutional Animal Use Protocol Committee at MacKay Medical College, Taiwan: A111003.

## 3 Illumination

Experimental procedures for LANs have been established and are widely used; however, there are some differences in the intensity, time, and duration of light delivery (Cissé et al., 2017; Mishra et al., 2019; An et al., 2020; Walker et al., 2020; Wu et al., 2021). Protocols were adapted appropriately for this study. Rats were first housed in a 06:00–18:00 12 h light/dark cycle (∼200 lux white ambient illumination at the cage level, light-emitting diode, LED) for at least seven days before starting habituation experiments. For the LANs, white (∼400 lux at the cage level, 380–780 nm, LED) or blue (∼400 lux at the cage level, 465–475 nm, LED) illumination was delivered from 19:00 to 22:00. Blue light at day (BLAD), blue light (∼400 lux at the cage level, 465–475 nm, LED) was delivered from 07:00 to 10:00. The LED array was placed above the cage. Light control rats were continuously maintained on a 12 h light/dark cycle (∼200 lux white ambient light during the day and no LAN). The illuminations were sustained for five days. Behavioral, electrophysiological, and immunoassay tests were conducted the day after or seven days after the end of LAN.

### 3.1 Resident-intruder test

The resident-intruder test (RIT) was performed, as described in our previous studies (Ye et al., 2019; Chou, 2020b, 2020a). Three of four group-housed rats were removed from their cages. One hour later, an unfamiliar intruder of similar weight was placed in the home cage for 10 min. Aggression was defined as any one of a number of events in which grooming (resident's forepaws compressing the intruder), chasing (resident chasing intruder), remaining offensively upright and holding the intruder down (resident upright and intruder down), and lateral threat behaviors were displayed by the resident. Sudden and unavoidable biting events during the RIT were calculated. The rats were separated using a plastic shield when the biting behavior was sustained for more than 2 s to minimize suffering. No bleeding injuries were observed in any of the intruders during the experiment. Biting behavior was defined as the number of events in which the resident broke or scratched the skin of the tail or body of the intruder with their teeth. The tests were conducted and scored by an experimenter who was blinded to the treatment conditions.

Previous studies have used foot shock as an acute stressor to trigger aggressive outbursts (referred to as impulsive aggression) (Chang et al., 2015; Chang and Gean, 2019). A mild intensity foot-shock stress was used in the present study that did not alter aggressive behavior in control rats, but did alter aggressive behaviors in night-time light exposure groups. Resident rats were placed in a chamber with a foot-shock grid (Panlab-Harvard Apparatus, Barcelona, Spain), and they received five foot shocks at 0.1 mA (1 s, at random intervals) 30 min before RIT.

### 3.2 Elevated plus-maze test

The elevated plus-maze test (EPMT) was used as an index of anxiety-like behavior and was performed as described in our previous study (Ye et al., 2019; Ko et al., 2020). Rats were adapted to the environment of the behavioral laboratory for at least 1 h per day for 3 days. On the test day, rats were carefully transferred to the central platform through the open arms for 15 min. Activity was collected by following the animal movement track using the EthoVision XT version 17 animal behavioral tracking system (Noldus, Wageningen, The Netherlands). The recorded time spent in the open arms was calculated as an index of anxiety.

### 3.3 Open field test

The open field test (OFT) was used as an index of anxiety-like behavior and an index of locomotor activity and was conducted according to our previous study (Ye et al., 2019; Ko et al., 2020; Li et al., 2023). Rats were first allowed to adapt to the environment of the behavioral laboratory for at least 1 h per day for 3 days. On the test day, rats were placed individually in the center of the apparatus (50 cm × 50 cm, 40 cm high) for 30 min. Activity was collected by following the animal's movement tracks using the EthoVision XT version 17 animal behavioral tracking system (Noldus, Wageningen, The Netherlands). The recorded time spent in the central zone (20 cm × 20 cm) was calculated as an index of anxiety. The total distance traveled was calculated as an index of locomotor activity.

### 3.4 Extracellular electrophysiology

Extracellular electrophysiological recordings for field excitatory postsynaptic potential (fEPSP) in the BLA were adapted and conducted as described previously (Ye et al., 2022; Xu et al., 2023). The brains of the rats were removed and sectioned, and BLA-containing coronal slices (400 μm-thick) were then equilibrated in aCSF at room temperature for at least 1 h before recording. The aCSF contained (in mM): 117 NaCl, 4.7 KCl, 2.5 CaCl_2_, 1.2 MgCl_2_, 1.2 NaH_2_PO_4_, 25 NaHCO_3_ and 11 D-glucose, which was bubbled continuously with 95% O_2_–5% CO_2_ to maintain pH at 7.4. To facilitate the recordings, 1 μM (+)-bicuculline was added to the aCSF solution.

For recording, a slice was transferred to a recording chamber, placed on a nylon net, and stabilized with a section of platinum filament. The slice was fully submerged and perfused with oxygenated aCSF. The solution flow rate was 2–3 mL/min. All experiments were carried out at 32 ± 1 °C. Extracellular recordings of the fEPSPs were obtained using microelectrodes filled with 3 M NaCl (resistance 1−5 MΩ). A bipolar stainless steel stimulating electrode was placed in the external capsule, and a capillary glass recording electrode (Harvard Apparatus, Holliston, MA, USA) was placed in the BLA. The intensity of the stimulus pulse (150 μs in duration) was set to elicit approximately 50% of the maximum fEPSP at a frequency of 0.05 Hz. The baseline fEPSPs were monitored for 10 min before the experiments. High-frequency stimulation (HFS) consisted of using a train (1 s) of stimuli at 100 Hz to evoke long-term potentiation (LTP). Low-frequency stimulation (LFS) consisted of 900 training sessions of stimuli at 1 Hz (1 s at 1-min intervals) for 15 min to induce long-term depression (LTD). The maintenance of LTP and LTD were calculated as the averaged changes in the fEPSP slope measured 60 min after HFS and LFS. The input‒output (IO) curve was evoked by stimulus intensities from 5 V to 15 V. The paired-pulse ratio (PPR) was evoked by two identical intensity stimuli at 10, 30, 40, 50, 100, and 200 ms interstimulation intervals. The second response was normalized to the first response. The strength of synaptic transmission was quantified by measuring the slope of the fEPSP. The fEPSP slope was measured from the linear part of the rising phase. Evoked fEPSPs were recorded using an AxoClamp-2B amplifier (Axon Instruments, Forster City, CA, USA) with an active bridge balance circuit. The evoked fEPSPs were collected, stored, and analyzed on an IBM-compatible computer with pClamp6.0 software (Axon Instruments, Foster City, CA, USA).

### 3.5 Immunoassay

The protein levels of total BDNF (#CYT306, Merk, Rahway, NJ, USA), mature BDNF (mBDNF) (#BEK-2211, Biosensis Pty Ltd, Thebarton, South Australia), pro-form BDNF (proBDNF) (#BEK-2237, Biosensis Pty Ltd, Thebarton, South Australia), phosphorylated tyrosine receptor kinase B (p-TrkB) (pan p-Tyr, #7108, Cell Signaling, Danvers, MA, USA), and total tyrosine receptor kinase B (TrkB) (#BEK-2338, Biosensis Pty Ltd, Thebarton, South Australia) were measured by commercialized enzyme-linked immunosorbent assay (ELISA) kits according to the standard protocol provided by the manufacturers. Briefly, the tissue samples of the BLA obtained from rats were weighed and then homogenized in ice-cold lysis Tris-HCl buffer solution (TBS; pH 7.4), and the homogenates were centrifuged for 20 min at 15,000×*g* at 4 °C. The levels of protein were calculated from regression analysis of a standard curve. Each value was normalized to the average of the control groups.

### 3.6 Brain cannula implantation and microinjection

The standard procedure was performed according to our previous studies (Chou, 2020a; Xu et al., 2023). Rats were first anesthetized with isoflurane (5% for induction and 2% for maintenance) and placed in a stereotaxic frame (Stoelting, Wood Dale, IL, USA). A 24-gauge, 12-mm stainless steel guide cannula was implanted stereotaxically into the BLA (AP: 2.8 mm; L: ±4.8 mm; DV: 7.5 mm) bilaterally. Rats were allowed to recover for at least 7 days before starting experiments. For microinjection, drugs were microinfused into the BLA bilaterally through a 30-gauge internal injection cannula via a 1 μl Hamilton syringe connected to a microinfusion pump (KDS311; KD Scientific Inc., Holliston, MA, USA). The drug solution at 0.2 μl was slowly infused into the BLA for 2 min, and an additional 5 min was allowed to prevent backflow of the drug. At the end of the microinjection experiment, the rats were sacrificed by perfusion, and the injection sites were evaluated for each animal. Only those rats that had accurate injections located in the BLA were chosen for analysis.

### 3.7 BDNF RNAi

The RNA interference (RNAi) sequence targeting BDNF mRNA (Dharmacon, Lafayette, CO, USA) and a scrambled control sequence (Dharmacon, Lafayette, CO, USA) were designed and used as previously described (Baker-Herman et al., 2004; Chou, 2020a). Four duplexes of the 21-nucleotide sequence with symmetrical 3’ overhangs were located within the BDNF RNAi. The sequences were 1) TCGAAGAGCTGCTGGATGA, 2) TATGTACACTGACCATTAA, 3) GAGCGTGTGTGACAGTATT, and 4) GAACTACCCAATCGTATGT. BDNF RNAi and scrambled RNAi were suspended and dissolved in RNAi Universal Buffer (Dharmacon, Lafayette, CO, USA) at 50 μM as a stock solution, which was aliquoted and stored at −20 °C. Prior to the intra-BLA microinjection experiments, BDNF RNAi or scrambled RNAi (200 nM/3.5 μl) was mixed with Oligofectamine (0.5 μl; Thermo Fisher Scientific, Waltham, MA, USA) and incubated at room temperature for 15 min. For behavioral experiments, BDNF RNAi (0.2 μl per side) or scrambled RNAi was microinfused 3.5 h before the RIT. The effects of BDNF RNAi and scrambled RNAi were examined by assessment of total BDNF protein levels (#CYT306, Merk, Rahway, NJ, USA) in the BLA at the end of behavioral experiments.

### 3.8 Reagents and vehicles

(−) Bicuculline methiodide (#2503, Tocris Bioscience, Ellisville, MO, USA), neutralizing BDNF antibody (nAB; #G164A, Promega, Madison, WI, USA; Merk Boston, MA, USA), sheep IgG (vehicle for BDNF nAB; #sc-2717, Santa Cruz, Dallas, TX, USA), K252a (#05288, Sigma‒Aldrich, St. Louis, MO, USA), rapamycin (#1292, Tocris Bioscience, Ellisville, MO, USA), recombinant BDNF (#G149A, Promega, Madison, WI, USA; #GF026, Merk, Boston, MA, USA), and 7,8-dihydroxyflavone (7,8-DHF, #3826, Tocris Bioscience, Ellisville, MO, USA) dissolved in water or dimethyl sulfoxide (DMSO) were used in this study. The final concentration of DMSO was controlled to less than 0.1% of water. The dose of each drug administered was based on our previous study (Chou, 2020a), and the dose of 7,8-DHF used was based on the previous study described (Sun et al., 2023).

### 3.9 Data analysis

The results in the study were analyzed using Prism 8 software (GraphPad software, San Diego, CA, USA) and expressed as the mean ± standard error of the mean (SEM). Data with one factor were analyzed by one-way analysis of variance (ANOVA), and data with two factors were analyzed by two-way ANOVA or repeated-measures two-way ANOVA. Bonferroni's post hoc analyses were used to compare group means. Significance was set at p < 0.05.

## 4 Results

### 4.1 BLAN produces mild shock stress-induced heightened aggression

**Fig. 1:**
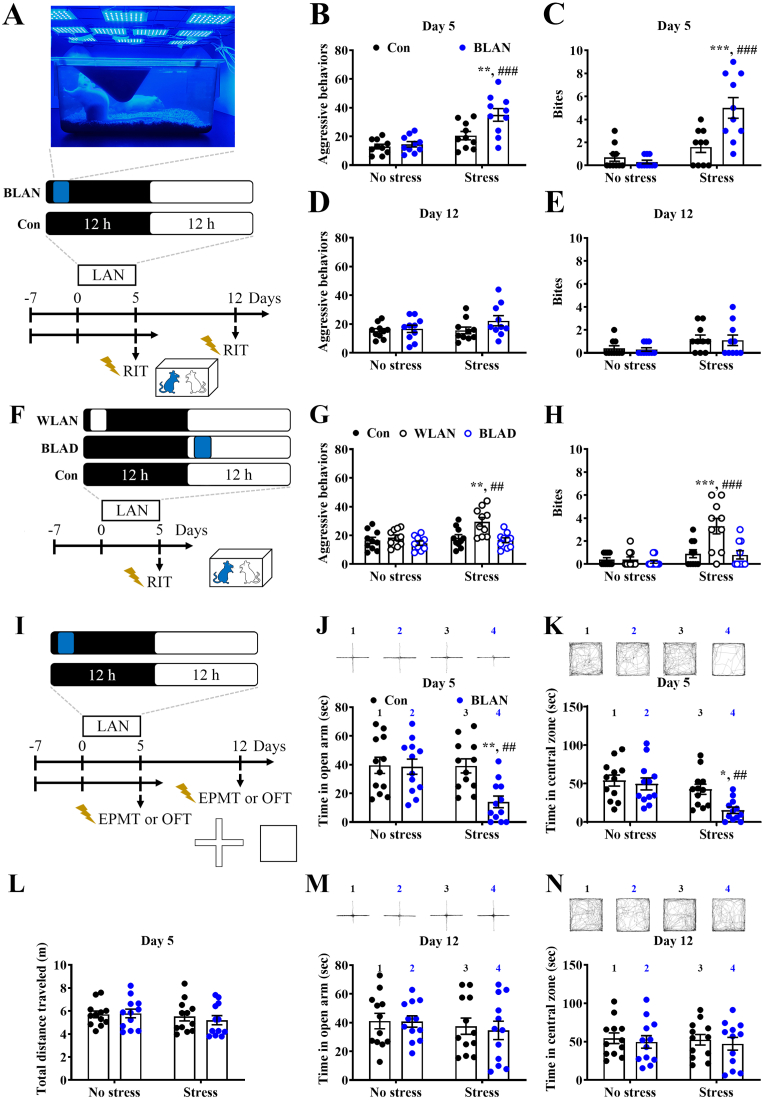
**Light at night results in shock stress-induced heightened aggression and anxiety-like behavior.** (A) Schematic illustrating the experimental design and procedure used for study of the effects of blue light at night (BLAN) on mild shock stress-induced aggression tested by the resident-intruder test (RIT) on Day 5 after illumination and the 7-day recovery on Day 12. Rats were habituated to a normal 12 h light/dark cycle for at least 7 days before the experiments. Example image showing the laboratory rat model of experimental blue illumination at night. (B–E) Scatterplots depicting the distributions of the events of aggressive behaviors of BLAN and control (Con) rats on Day 5 (B) and on Day 12 (D) and the number of bites on Day 5 (C) and on Day 12 (E). (B: Stress effect: F_(1,36)_ = 23.75, p < 0.0001, BLAN effect: F_(1,36)_ = 7.797, p = 0.0083, interaction: F_(1,36)_ = 5.006, p = 0.0315; C: Stress effect: F_(1,36)_ = 26.98, p < 0.0001, BLAN effect: F_(1,36)_ = 7.744, p = 0.0085, interaction: F_(1,36)_ = 12.42, p = 0.0012; D: Stress effect: F_(1,36)_ = 1.351, p = 0.2527, BLAN effect: F_(1,36)_ = 2.523, p = 0.1209, interaction: F_(1,36)_ = 1.015, p = 0.3205; E: Stress effect: F_(1,36)_ = 6.227, p = 0.0173, BLAN effect: F_(1,36)_ = 0.0973, p = 0.7569, interaction: F_(1,36)_ = 1.499e-031, p > 0.9999, n = 10 in each group, two-way ANOVA). (F) Schematic illustrating the experimental protocol used for examination of the effects of white light at night (WLAN) and blue light at day (BLAD) on stress-induced aggression on Day 5. (G and H) Scatterplots depicting the distributions of the aggressive (G) and biting events (H) on Day 5. (G: Stress effect: F_(1,54)_ = 8.179, p = 0.006, BLAN effect: F_(2,54)_ = 8.764, p = 0.0005, interaction: F_(2,54)_ = 3.093, p = 0.0535; H: Stress effect: F_(1,54)_ = 20.28, p < 0.0001, BLAN effect: F_(2,54)_ = 8.328, p = 0.0007, interaction: F_(2,54)_ = 7.01, p = 0.002, n = 10 in each group, two-way ANOVA). (I) Schematic illustrating the experimental protocol used for investigation of the effects of BLAN on mild shock stress-induced anxiety-like behavior tested by the elevated plus maze test (EPMT) and open field test (OFT) on Day 5 and recovery Day 12. (J–N) Example of tracking traces and scatterplots depicting the distributions of the time spent in the open arm in the EPMT on Day 5 (J) and Day 12 (M), the time spent in the central zone in the OFT on Day 5 (K) and Day 12 (N), and the total distance traveled in the OFT on Day 5 (L). (J: Stress effect: F_(1,44)_ = 6.238, p = 0.0163, BLAN effect: F_(1,44)_ = 6.825, p = 0.0123, interaction: F_(1,44)_ = 5.874, p = 0.0195; K: Stress effect: F_(1,44)_ = 12.28, p = 0.0011, BLAN effect: F_(1,44)_ = 5.799, p = 0.0203, interaction: F_(1,44)_ = 3.043, p = 0.0881; L: Stress effect: F_(1,44)_ = 1.08, p = 0.3043, BLAN effect: F_(1,44)_ = 0.1166, p = 0.7344, interaction: F_(1,44)_ = 0.3151, p = 0.5774; M: Stress effect: F_(1,44)_ = 0.8138, p = 0.3719, BLAN effect: F_(1,44)_ = 0.08329, p = 0.7742, interaction: F_(1,44)_ = 0.05746, p = 0.8117; N: Stress effect: F_(1,44)_ = 0.08067, p = 0.7777, BLAN effect: F_(1,44)_ = 0.4362, p = 0.5124, interaction: F_(1,44)_ = 0.0006002, p = 0.9806, n = 12 in each group, two-way ANOVA). Data represent mean ± SEM. *P < 0.05, **P < 0.01 and ***P < 0.001 compared with Con + Stress, ^##^*P* < 0.01, and ^###^*P* < 0.001 compared with BLAN + No stress, by Bonferroni's post-hoc test. (For interpretation of the references to colour in this figure legend, the reader is referred to the Web version of this article.)

We first established a night-illuminated rat model (Fig. 1A and F). A 5-day BLAN exposure did not alter the elements of aggressive behavior (Fig. 1B) or biting events (Fig. 1C), but increased mild shock stress-evoked aggressive (Fig. 1B) and elevated stress-evoked biting (Fig. 1C) behaviors. Interestingly, these effects were not observed after a 7-day recovery period from BLAN (Fig. 1D and E), indicating that the effects of BLAN on behavior were reversible. In addition to BLAN, white light at night (WLAN), but not BLAD, resulted in stress-evoked heightened aggressive (Fig. 1G) and increased stress-evoked biting (Fig. 1H) behaviors. These behavioral results suggest that nocturnal blue light exposure produce stress-evoked heightened aggression in a time-dependent and reversible manner, but has no effect on basal aggression.

Aggression was associated with anxiety phenotype (Rosell and Siever, 2015). Additionally, LAN may influence anxiety-like behaviors (Borniger et al., 2014; Cissé et al., 2016). We examined the effects of BLAN on anxiety-like behaviors. BLAN was also shown to reduce the time that stressed rats spent in the open arm in the EPMT (Fig. 1I and J) and decrease the time that stressed rats spent in the central zone in the OFT (Fig. 1I and K); however, BLAN did not change the total distance traveled in the OFT (Fig. 1L). Similarly, these effects of BLAN did not appear after a 7-day recovery from BLAN (Fig. 1I, M, and 1N). These results suggest that nocturnal blue light increases stress-evoked anxiety-like behavior in a reversible manner.

### 4.2 BLAN elevates synaptic plasticity and BDNF expression and signaling in the BLA

**Fig. 2:**
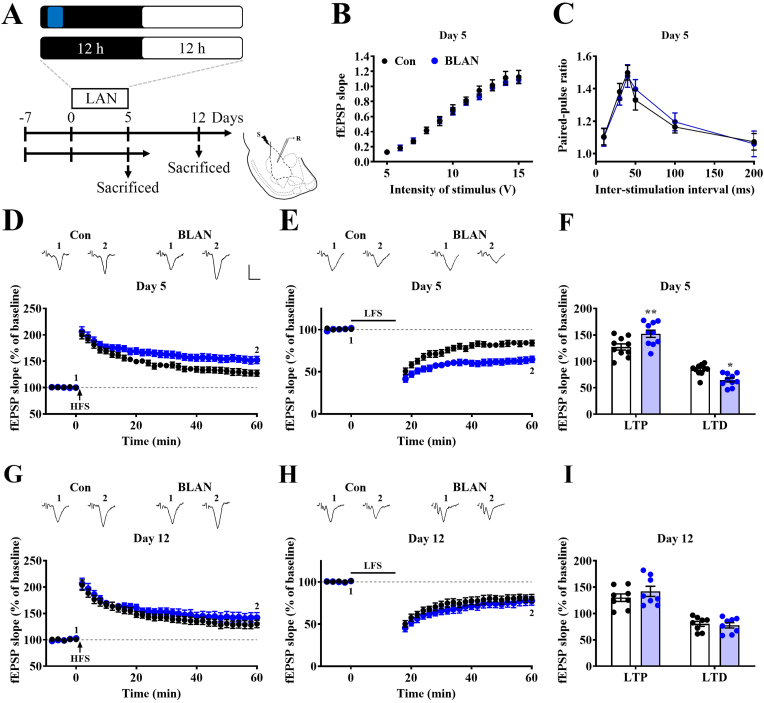
**BLAN potentiates synaptic plasticity in the basolateral amygdala.** (A) Schematic illustrating the experimental procedure used for investigation of the synaptic effects of BLAN on the basolateral nucleus of the amygdala (BLA) on Day 5 after illumination and recovery on Day 12. Histology of the BLA illustrating the positions of the stimulating (S) electrode in the external capsule and recording (R) electrode in the BLA. (B) Summary of experiments indicating the input‒output curve in the BLA obtained from BLAN and Con rats. (intensity effect: F_(10,180)_ = 238.7, p < 0.0001; BLAN effect: F_(1,18)_ = 0.1672, p = 0.6875; interaction: F_(10,180)_ = 0.3748, p = 0.9563, n = 10 in each group, 10 slices obtained from 10 rats, repeated-measures two-way ANOVA). (C) Summary of experiments indicating the paired-pulse ratio (PPR) in the BLA obtained from BLAN and Con rats. (interval effect: F_(5,70)_ = 17.42, p < 0.0001; BLAN effect: F_(1,14)_ = 0.01711, p = 0.8978; interaction: F_(5,70)_ = 0.2413, p = 0.9428, n = 8 in each group, 8 slices obtained from 8 rats, repeated-measures two-way ANOVA). (D and E) Upper inset: representative recording traces of field excitatory postsynaptic potentials (fEPSPs) at baseline (1) and at 60 min (2) after high-frequency stimulation (HFS)-induced long-term potentiation (LTP) (D) and low-frequency stimulation (LFS)-induced long-term depression (LTD) (E) in the BLA obtained from BLAN and Con rats. Calibration: 0.5 mV, 5 ms. Lower inset: summary of experiments indicating HFS-evoked LTP (D) and LFS-evoked LTD (E) on Day 5. (D: time effect: F_(34,612)_ = 142.6, p < 0.0001; BLAN effect: F_(1,18)_ = 5.444, p = 0.0314; interaction: F_(34,612)_ = 4.489, p < 0.0001; E: time effect: F_(3.712,66.81)_ = 122, p < 0.0001; BLAN effect: F_(1,18)_ = 15.94, p = 0.0009; interaction: F_(26,468)_ = 8.584, p < 0.0001, n = 10 in each group, 10 slices obtained from 10 rats were recorded, analyzed by repeated-measures two-way ANOVA). (F) Scatterplots indicating LTP (after HFS for 60 min) and LTD (after LFS for 60 min) in the BLAN and Con on Day 5. (plasticity effect: F_(1,36)_ = 160, p < 0.0001, BLAN effect: F_(1,36)_ = 0.2862, p = 0.596, interaction: F_(1,36)_ = 18.46, p = 0.0001, n = 10 in each group, two-way ANOVA). (G and H) Summary of experiments and representative recording traces of fEPSPs after HFS-induced LTP (G) and LFS-induced LTD (H) on Day 12. (G: time effect: F_(34,476)_ = 1, p = 0.4704; BLAN effect: F_(1,14)_ = 1.003, p = 0.3336; interaction: F_(34,476)_ = 1, p = 0.4711; H: time effect: F_(2.335,32.68)_ = 75.88, p < 0.0001; BLAN effect: F_(1,14)_ = 0.8357, p = 0.3761; interaction: F_(26,364)_ = 0.5893, p = 0.9475, n = 8 in each group, 8 slices obtained from 8 rats were recorded, analyzed by repeated-measures two-way ANOVA). (F) Scatterplots indicating LTP (after HFS for 60 min) and LTD (after LFS for 60 min) in the BLAN and Con groups on Day 12. (plasticity effect: F_(1,28)_ = 68.18, p < 0.0001, BLAN effect: F_(1,28)_ = 0.4351, p = 0.5149, interaction: F_(1,28)_ = 1.136, p = 0.2956, n = 8 in each group, two-way ANOVA). Data represent mean ± SEM. *P < 0.05 and **P < 0.01 compared with Con.

The BLA is believed to play a role in the regulation of emotions, including aggression and anxiety (Rosell and Siever, 2015; Blair, 2016). Synaptic plasticity in the BLA may also be associated with stress-induced responses (Rattiner et al., 2004; Chang et al., 2015; Chang and Gean, 2019). We focused on the BLA and explored the potential impact of the BLAN on synaptic activity (Fig. 2A). The BLAN had no significant influence on the input‒output curve (Fig. 2B) or paired-pulse facilitation (Fig. 2C). Thus, BLAN elevated LTP (Fig. 2D and F) and LTD (Fig. 2E and F). However, LTP and LTD enhancements did not appear after a 7-day recovery period from BLAN (Fig. 2A, G, 2H, and 2I). These results suggest that night-time blue light exposure elevates synaptic plasticity in the BLA in a reversible manner.

**Fig. 3:**
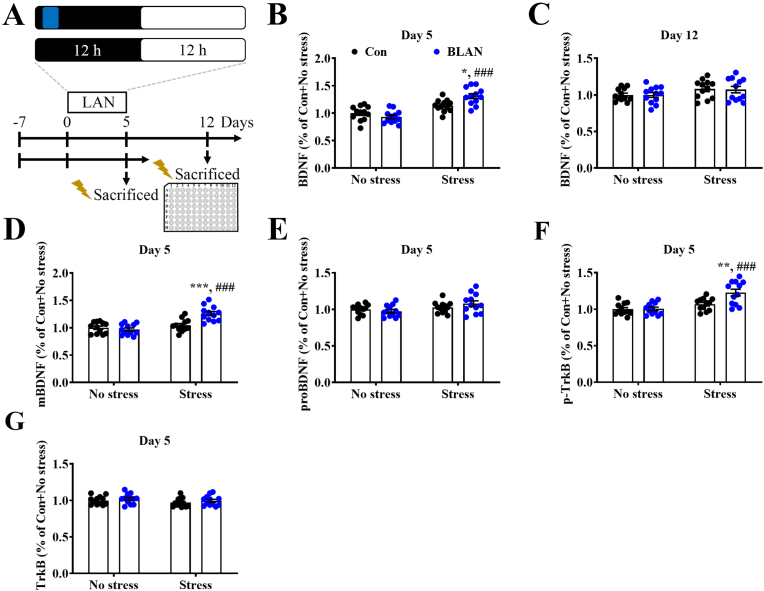
**BLAN produces stress-induced increased BDNF expression and signaling in the BLA.** (A) Schematic illustrating the experimental procedure used for exploration of the effects of BLAN on the brain-derived neurotrophic factor (BDNF) level in the BLA on Day 5 after illumination and recovery on Day 12. (B and C) Scatterplots depicting the distributions of the BDNF protein level in the BLA obtained from BLAN and Con rats on Day 5 (B) and on Day 12 (C). (B: Stress effect: F_(1,44)_ = 48.32, p < 0.0001, BLAN effect: F_(1,44)_ = 2.106, p = 0.1538, interaction: F_(1,44)_ = 10.6, p = 0.0022; C: Stress effect: F_(1,44)_ = 4.849, p = 0.0329, BLAN effect: F_(1,44)_ = 0.01661, p = 0.8981, interaction: F_(1,44)_ = 0.01387, p = 0.9068, n = 12 in each group, two-way ANOVA). (D–G) Scatterplots depicting the distributions of the mature form BDNF (mBDNF) (D), pro-form BDNF (E), phosphorylated tyrosine receptor kinase B (p-TrkB) (F), and total form TrkB (G) levels in the BLA obtained from BLAN and Con rats on Day 5. (D: Stress effect: F_(1,44)_ = 26.46, p < 0.0001, BLAN effect: F_(1,44)_ = 7.636, p = 0.0083, interaction: F_(1,44)_ = 14.05, p = 0.0005; E: Stress effect: F_(1,44)_ = 5.871, p = 0.0196, BLAN effect: F_(1,44)_ = 0.2531, p = 0.6174, interaction: F_(1,44)_ = 2.131, p = 0.1515; F: Stress effect: F_(1,44)_ = 22.54, p < 0.0001, BLAN effect: F_(1,44)_ = 7.241, p = 0.01, interaction: F_(1,44)_ = 6.234, p = 0.0164; F: Stress effect: F_(1,44)_ = 2.136, p = 0.151, BLAN effect: F_(1,44)_ = 1.407, p = 0.242, interaction: F_(1,44)_ = 0.02492, p = 0.8753, n = 12 in each group, two-way ANOVA). Data represent mean ± SEM. *P < 0.05, **P < 0.01, and ***P < 0.001 compared with Con + Stress, ^###^*P* < 0.001 compared with BLAN + No stress, by Bonferroni's post-hoc test.

BDNF and its signaling pathways play critical roles in the regulation of synaptic plasticity, including LTP and LTD (Andero et al., 2014). We examined the influence of BLAN exposure on BDNF expression in the BLA (Fig. 3A). BLAN appeared to elevate BDNF levels in stressed rats (Fig. 3B), but not after a 7-day recovery from BLAN (Fig. 3C). Moreover, BLAN increased mBDNF expression (Fig. 3D), but did not significantly alter proBDNF levels (Fig. 3E) in stressed rats. In addition, BLAN increased the expression of phosphorylated TrkB (Fig. 3F), but did not significantly change the total form of TrkB (Fig. 3G). Collectively, these data suggest that nocturnal blue light exposure results in the stress-induced elevation of BDNF and its signaling in the BLA in a reversible manner.

### 4.3 BDNF in the BLA is involved in stress-induced heightened aggression in BLAN rats

**Fig. 4:**
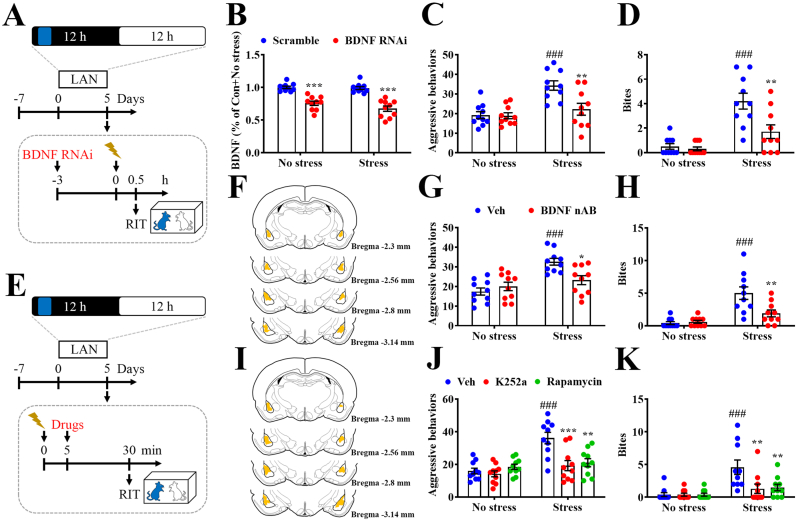
**Inhibition of BDNF signaling in the BLA blocks stress-induced heightened aggression in BLAN rats.** (A) Schematic illustrating the experimental procedure used for investigation of the effects of intra-BLA application of BDNF RNAi (0.2 μl per side) and scramble (0.2 μl per side) on stress-induced aggression in BLAN rats. (B) Scatterplots depicting the distributions of BDNF levels in the BLA. (B: Stress effect: F_(1,36)_ = 2.438, p = 0.1272, BDNF RNAi effect: F_(1,36)_ = 85.89, p < 0.0001, interaction: F_(1,36)_ = 1.428, p = 0.2399, n = 10 in each group, two-way ANOVA). (C and D) Scatterplots depicting the distributions of the events of aggressive behaviors (C) and the number of bites (D). (C: Stress effect: F_(1,36)_ = 16.61, p = 0.0002, BDNF RNAi effect: F_(1,36)_ = 7.545, p = 0.0093, interaction: F_(1,36)_ = 6.832, p = 0.013; D: Stress effect: F_(1,36)_ = 32.38, p < 0.0001, BDNF RNAi effect: F_(1,36)_ = 9.075, p = 0.0047, interaction: F_(1,36)_ = 6.585, p = 0.0146, n = 10 in each group, two-way ANOVA). (E) Schematic illustrating the experimental protocol used to study the effects of intra-BLA application of BDNF neutralizing antibody (nAB, 0.2 μg/0.2 μl per side), K252a (10 pM/0.2 μl per side), and rapamycin (100 μM/0.2 μl per side) on stress-induced aggression in BLAN rats. (F) Image showing the histology of the BLA and the summarized injection area of the BDNF nAB and vehicle (Veh, sheep IgG; 0.2 μg/0.2 μl per side). (G and H) Scatterplots depicting the distributions of aggressive events (G) and bites (H). (G: Stress effect: F_(1,36)_ = 20.72, p < 0.0001, BDNF nAB effect: F_(1,36)_ = 2.83, p = 0.1012, interaction: F_(1,36)_ = 8.814, p = 0.0053; H: Stress effect: F_(1,36)_ = 26.62, p < 0.0001, BDNF nAB effect: F_(1,36)_ = 6.431, p = 0.0157, interaction: F_(1,36)_ = 8.327, p = 0.0066, n = 10 in each group, two-way ANOVA). (I) BLA histology showing the injection area of K252a, rapamycin, and Veh. (J and K) Scatterplots depicting the distributions of the aggressive (J) and biting events (K). (J: Stress effect: F_(1,54)_ = 20.78, p < 0.0001, drugs effect: F_(2,54)_ = 7.304, p = 0.0016, interaction: F_(2,54)_ = 7.455, p = 0.0014; K: Stress effect: F_(1,54)_ = 17.81, p < 0.0001, drugs effect: F_(2,54)_ = 4.76, p = 0.0125, interaction: F_(2,54)_ = 4.76, p = 0.0125, n = 10 in each group, two-way ANOVA). Data represent mean ± SEM. *P < 0.05, **P < 0.01, and ***P < 0.001 compared with Scramble in (B) or Veh + Stress, ^###^*P* < 0.001 compared with Scramble + No stress or Veh + No stress, by Bonferroni's post-hoc test.

We then investigated the role of BDNF and its signaling in the stress-induced enhancement of aggression in BLAN rats (Fig. 4A and E). Intra-BLA microinfusion (Sup. Fig. 1A) of RNA interference targeting BDNF downregulated BDNF levels in the BLA (Fig. 4B), and decreased the stress-induced elevation of aggressive elements (Fig. 4C) and biting behavior (Fig. 4D). Moreover, intra-BLA microinjection of BDNF nAB (Fig. 4F) attenuated the stress-induced increase in aggressive (Fig. 4G) and biting events (Fig. 4H). Similarly, intra-BLA application of the selective TrkB inhibitor K252a and mTOR inhibitor rapamycin (Fig. 4I) blocked the stress-induced elevated behavioral events of aggression (Fig. 4J) and biting (Fig. 4K). These behavioral data implicate the inhibition of the expression, release, and signaling of BDNF in the BLA, which blocks the stress-induced heightened aggression in BLAN rats.

**Fig. 5:**
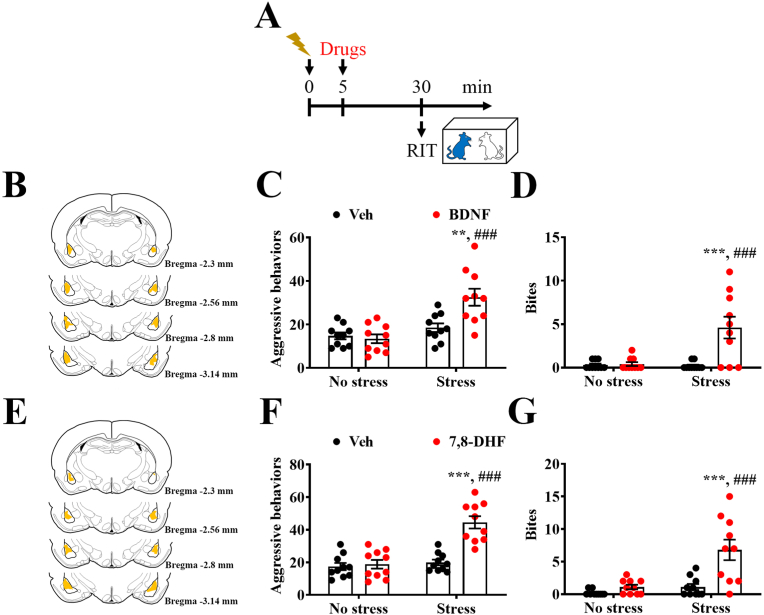
**Activation of BDNF signaling in the BLA results in stress-evoked heightened aggression.** (A) Schematic illustrating the experimental procedure used to study the effects of intra-BLA application of BDNF (0.2 ng/0.2 μl per side) and 7,8-dihydroxyflavone (7,8-DHF, 1 μg/0.2 μl per side) on shock stress-induced aggression in naïve rats. (B) Image of BLA histology showing the injection area of BDNF and Veh (0.2 μl per side). (C and D) Scatterplots depicting the distributions of the aggressive events (C) and the number of bites (D). (C: Stress effect: F_(1,36)_ = 20.58, p < 0.0001, BDNF effect: F_(1,36)_ = 6.329, p = 0.0165, interaction: F_(1,36)_ = 9.186, p = 0.0045; D: Stress effect: F_(1,36)_ = 10.19, p = 0.0029, BDNF effect: F_(1,36)_ = 12.27, p = 0.0012, interaction: F_(1,36)_ = 11.21, p = 0.0019, n = 10 in each group, two-way ANOVA). (E) BLA histology showing the injection area of 7,8-DHF and Veh (0.2 μl per side). (F and G) Scatterplots depicting the distributions of the aggressive events (F) and the number of bites (G). (F: Stress effect: F_(1,36)_ = 27.01, p < 0.0001, 7,8-DHF effect: F_(1,36)_ = 22.96, p < 0.0001, interaction: F_(1,36)_ = 18.28, p = 0.0001; G: Stress effect: F_(1,36)_ = 15.37, p = 0.0004, BDNF effect: F_(1,36)_ = 15.37, p = 0.0004, interaction: F_(1,36)_ = 8.132, p = 0.0072, n = 10 in each group, two-way ANOVA). Data represent mean ± SEM. **P < 0.01 and ***P < 0.001 compared with Veh + Stress, ^###^*P* < 0.001 compared with BDNF + No stress or 7,8-DHF + No stress, by Bonferroni's post-hoc test.

In contrast to the inhibition of BLA BDNF, intra-BLA application of BDNF (Fig. 5B) resulted in a stress-induced increase in aggressive elements (Fig. 5A and C) and biting behavior (Fig. 5A and D) in naïve rats. In addition, intra-BLA application of the selective TrkB agonist 7,8-DHF (Fig. 5E) produced stress-induced enhancement of aggressive elements (Fig. 5A and F) and biting behavior (Fig. 5A and G). The results indicate that BDNF and signaling activation in the BLA produce stress-induced heightened aggression.

## 5 Discussion

In modern cities, sunset is no longer considered a signal at the end of the day. However, studies have reported that nocturnal light pollution can induce negative moods (Bedrosian and Nelson, 2013). The impact of LAN on aggression remains unknown, and the underlying mechanisms are still largely unclear. In the present study, the results demonstrated that chronic BLAN exposure results in a stress-induced heightened aggressive phenotype, which could occur by enhancing BDNF expression and signaling in the BLA, and is associated with the potentiation of BLA synaptic plasticity. To the best of our knowledge, this is the first study to report the impact of BLAN on stress-provoked aggression and further provide novel insights into a BLA BDNF-dependent underlying mechanism. This study revealed that BLAN could be a potential risk factor for aggression in modern human society.

Exposure to night-time light is common in modern industrialized countries (Falchi et al., 2016; Walker et al., 2020). This includes artificial indoor light exposure at night, such as home lights turned on during the night and use of smartphones, tablets, television, and computer monitors, outdoor light exposure at night, such as road illumination; and night work and non-stop economic activities (Bedrosian and Nelson, 2013; Obayashi et al., 2013; Min and Min, 2018; Zielinska-Dabkowska, 2018; An et al., 2020; Walker et al., 2020). These nocturnal light exposures are less recognized in our daily lives. Nocturnal light pollution has been recognized as a major risk factor influencing human health, including obesity, breast cancer, and most importantly, mental health (Muscogiuri et al., 2022; Tancredi et al., 2022). Therefore, the over-illumination of individuals’ night-lives raises concerns about their potentially detrimental biological impacts, particularly on mood (Bedrosian and Nelson, 2013). Accumulating evidence indicates that exposure to LAN results in depressive-like behavior in rodents, as well as anxiety-like behavior (Borniger et al., 2014; An et al., 2020; Walker et al., 2020; Wu et al., 2021), and changes in many other phenotypes, such as reduced social preference and increased repetitive behaviors (Wang et al., 2020). These mood-relevant behavioral effects are likely mediated by the retinal melanipsin-expressing ganglion cell pathway (An et al., 2020). Selective ablation of the pathway blocks BLAN-mediated actions (An et al., 2020). The results of the present study suggest that BLAN not only increases stress-evoked anxiety-like behavior but also produces stress-evoked heightened aggressive behavior, providing a new direction for studying the impact of LAN on mood. Interestingly, an altered state of anxiety may lead to susceptibility to aggression (Rosell and Siever, 2015). Surprisingly, the present results showed that BLAN did not alter basal behavior relevant to aggression and anxiety, which may be due to the time, intensity, and duration of BLAN exposure used in this study. The effectiveness of light during the night phase in increasing stress-induced aggressive and anxiety-like behaviors was augmented. It is plausible that although light regulates activities and the sleep/wake cycle in opposite directions in diurnal versus nocturnal animals, when light appears during the wrong phase of the circadian rhythm, it may cause mood dysregulation in both nocturnal and diurnal animals (An et al., 2020).

The amygdala, including its subarea, the BLA, plays a critical role in the regulation of emotions and mental health, including post-traumatic stress disorder (PTSD), depression, anxiety, and aggression (Rosell and Siever, 2015; Blair, 2016; Prager et al., 2016; da Cunha-Bang and Knudsen, 2021; Zhang et al., 2023). Heightened reactivity of the amygdala to acute threatening stimuli is associated with an aggressive phenotype (Rosell and Siever, 2015). Most recently, a study indicated that short-wavelength LAN increased the number of c-fos-positive cells in the BLA (Wang et al., 2023). These results indicate the potential impact of BLAN on BLA activity and BLA-regulated behavioral performance. In the present study, the results demonstrated that mild foot shock stress elevated BLA BDNF levels and signaling in BLAN rats. Moreover, BLAN exposure enhanced synaptic plasticity in the BLA. In addition, the inhibition of BLA BDNF and its signaling blocks the effects of BLAN on stress-induced heightened aggression. Activation of BLA BDNF signaling mimics the effects of BLAN on stress-induced heightened aggression. These results support previous observations that BLAN alters BLA activity (Wang et al., 2023). Furthermore, the present results suggested that BLA BDNF plays a role in the action of BLAN on stress-induced heightened aggression. However, BDNF is involved in regulating synaptic plasticity (Andero et al., 2014). BDNF signaling and synaptic plasticity in the BLA are thought to be the molecular and cellular mechanisms of fear memory, and are associated with PTSD phenotypes (Andero et al., 2014). Therefore, the potential influence of BLAN on fear memory warrants further investigation. Interestingly, the PTSD animal model fear conditioning paradigm, using foot shock stress as a conditional stimulus, has been shown to increase BLA BDNF and signaling (Rattiner et al., 2004). It is likely that BLAN, which alters BLA BDNF levels and synaptic plasticity, is maladaptive to acute stress, leading to stress-provoked aggressive phenotypes. Different wavelengths of nocturnal light exposure may play distinct roles in the mood modulation. Short wavelengths of light, including blue light, primarily contribute to the effects of LAN, whereas long wavelengths reduce its negative effects (Wang et al., 2023). The present study showed that blue or white (containing blue light) LAN resulted in stress-induced enhancement of aggression, which may support the negative consequences of exposure to short wavelengths of light at night. The time and phase of light exposure may be other critical factors affecting mood. In this study, illumination was delivered from 19:00 to 22:00. Future studies should examine other durations of illumination. Moreover, the results showed that blue light exposure during the day phase did not alter basal or stress-evoked aggressive behavior. Regarding light intensity, dim LAN has been shown to negatively affect mood (Bedrosian et al., 2011; Cissé et al., 2016; Wang et al., 2023). The present study used approximately 400 lux light intensity, which is close to modern living room night-time light intensity (100–300 lux) (Gaston et al., 2013; Walker et al., 2020). Therefore, the effects of dim BLAN on aggression should be examined in future studies.

The present results showed that BLAN did not significantly alter basal synaptic transmission or basal levels of BDNF and signaling, which may be associated with the time and duration of light exposure used in this study. The results consistently showed that BLAN did not significantly change the basal behavioral phenotypes related to aggression and anxiety. The synergy between BLAN and acute stress on behavior, synaptic plasticity, and BDNF signaling may imply that BLAN is a potential risk factor.

The present results showed that a 5-day exposure to BLAN, but not after a 7-day re-exposure to a normal light-dark cycle, produced a stress-induced increase in aggressive behavior and BDNF expression and potentiated LTP and LTD. These results imply that the behavioral, synaptic, and molecular effects of BLAN are reversible, at least under the conditions used in this study.

The limitations of this study should be mentioned with careful consideration, particularly when applying the findings from the rodent study to a human context (An et al., 2020). First, using nocturnal rodent models to study the effects of LAN on mood is challenging. Whether the present outcomes of nocturnal rat studies correlate with those of night-time light exposure in diurnal humans remains unclear. Additionally, light is an aversive signal that can be a stressful stimulus for rodents (Barker et al., 2010). Moreover, this study was conducted only in male rats. Therefore, future studies should be performed in female rats. Furthermore, this study did not test the specific cell types involved in this phenomenon, such as neurons and glia. Future studies are required to address this issue. Finally, aggressive phenotypes in rodents are not characterized by negative or positive behavior. Aggression also represents adaptive behavior that occurs in the presence of an intruder (Nelson and Trainor, 2007). Therefore, to precisely understand the influence of nocturnal light exposure on human mood, human participants, including those with aggressive phenotypes and amygdala activity, should be tested in future studies.

In this post-industrial era, exposure to LAN has become increasingly inevitable. Studying its potential impact on mood and unraveling the underlying mechanisms are critical issues. The present study reported that nocturnal blue light exposure results in stress-evoked heightened aggression through the enhancement of BDNF in the BLA, which is associated with the potentiation of synaptic plasticity.

## CRediT authorship contribution statement

**Zhenlong Li:** Data curation, Formal analysis, Investigation, Methodology, Supervision, Validation, Visualization. **Chau-Shoun Lee:** Funding acquisition, Resources, Writing – review & editing. **Si Chen:** Methodology, Resources, Software, Supervision, Validation, Visualization. **Benyu He:** Methodology, Validation, Visualization. **Xinya Chen:** Methodology, Validation, Visualization. **Hsien-Yu Peng:** Writing – review & editing. **Tzer-Bin Lin:** Writing – review & editing. **Ming-Chun Hsieh:** Writing – review & editing. **Cheng-Yuan Lai:** Writing – review & editing. **Dylan Chou:** Conceptualization, Data curation, Formal analysis, Funding acquisition, Investigation, Methodology, Project administration, Resources, Software, Supervision, Validation, Visualization, Writing – original draft, Writing – review & editing.

## Declaration of competing interest

Authors declare that no conflict of interest.

## Data Availability

Data will be made available on request.
