# Supplementary material for: Blue light at night produces stress-evoked heightened aggression by enhancing brain-derived neurotrophic factor in the basolateral amygdala
Source: Neurobiol Stress. 2023 Dec 15;28:100600. doi: 10.1016/j.ynstr.2023.100600 (PMC10767493; doi:10.1016/j.ynstr.2023.100600)
Supplement: Multimedia component 1 [file mmc1.docx]

**Supplementary Results**

To check pharmacologically microinjection that targets the basolateral amygdala (BLA), eosin staining solution (#PH0500, Phygene, Fuzhou, Fujian, China) was used as a reporter and was microinfused (0.2 μl) into the BLA. The results showed that the reporter was expressed densely in the BLA (Sup Fig 1).

**
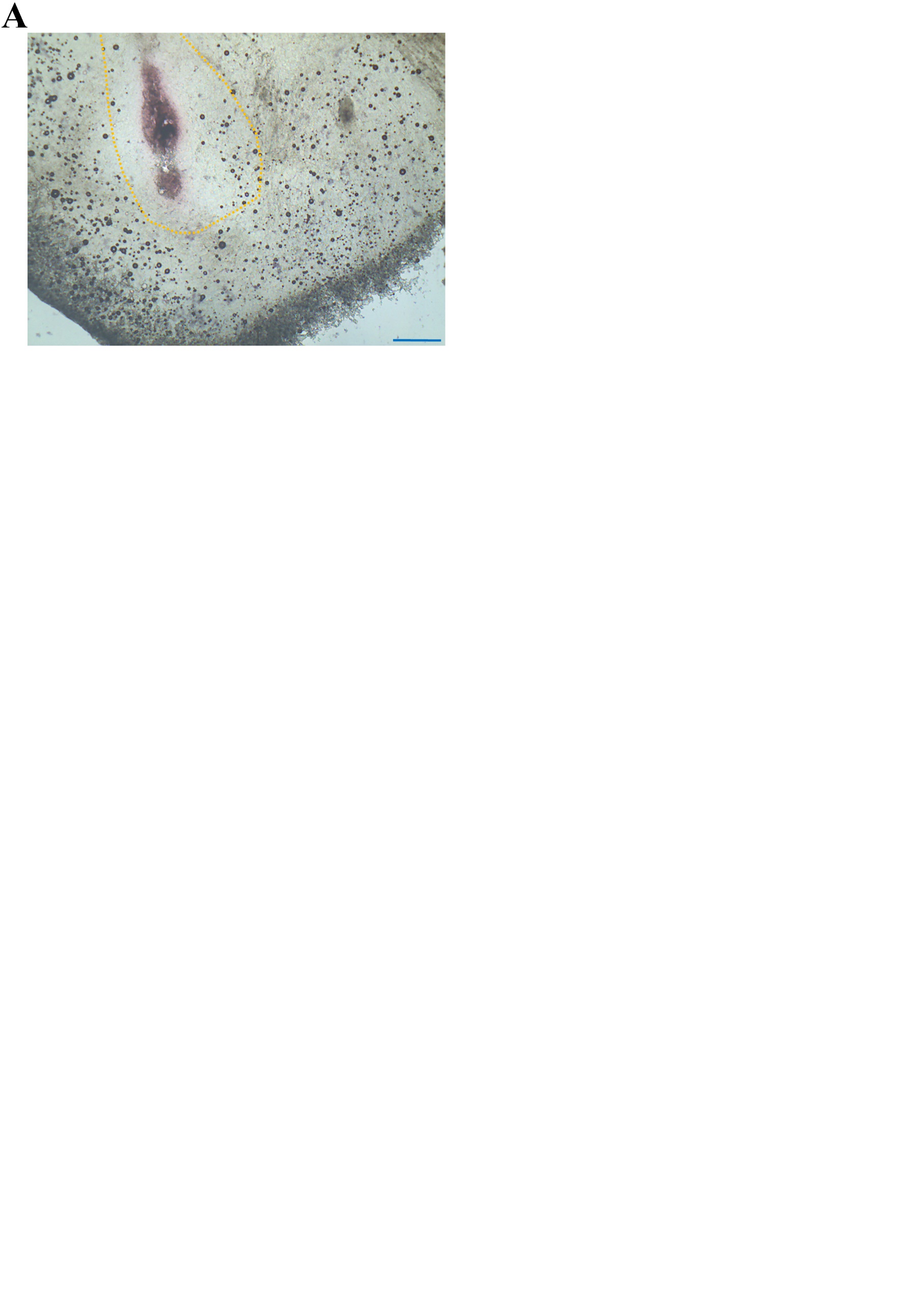
**

**Supplementary Figure 1. Diffusion of a microinjected reporter targeting the BLA.** (A) Example of a basolateral amygdala (BLA)-containing slice image illustrating the diffusion of an eosin staining solution (0.2 μl) microinjected into the BLA and conducted by Nissl staining (#G1430, Solarbio, Beijing, China). Scale bar in blue, 500 μm.
